# MAX deficiency impairs human endometrial decidualization through down-regulating OSR2 in women with recurrent spontaneous abortion

**DOI:** 10.1007/s00441-022-03579-z

**Published:** 2022-02-11

**Authors:** Weixu Ma, Mingzhu Cao, Shilei Bi, Lili Du, Jingsi Chen, Haibin Wang, Yufei Jiang, Yixuan Wu, Yixin Liao, Shuangbo Kong, Jianqiao Liu

**Affiliations:** 1 Department of Obstetrics and Gynecology, Center for Reproductive Medicine, Key Laboratory for Major Obstetric Diseases of Guangdong Province, The Third Affiliated Hospital of Guangzhou Medical University, Guangzhou, China; 2 Key Laboratory for Reproductive Medicine of Guangdong Province, The Third Affiliated Hospital of Guangzhou Medical University, Guangzhou, China; 3 Department of Obstetrics and Gynecology, Key Laboratory for Major Obstetric Diseases of Guangdong Province, The Third Affiliated Hospital of Guangzhou Medical University, Guangzhou, China; 4 Key Laboratory of Reproduction and Genetics of Guangdong Higher Education Institutes, Guangzhou, China; 5 Guangdong Engineering and Technology Research Center of Maternal-Fetal Medicine, Guangzhou, China; 6 Guangdong-Hong Kong-Macao Greater Bay Area Higher Education Joint Laboratory of Maternal-Fetal Medicine, Guangzhou, China; 7 Department of Obstetrics and Gynecology, The First Affiliated Hospital of Xiamen University, Xiamen University, Xiamen, China; 8 Fujian Provincial Key Laboratory of Reproductive Health Research, School of Medicine, Xiamen University, Xiamen, China; 9 Xiamen Key Laboratory of Reproduction and Genetics, Department of Reproductive Medicine, Women and Children’s Hospital, School of Medicine, Xiamen University, Xiamen, China; 10 Department of Obstetrics and Gynecology, Nanfang Hospital, Southern Medical University, Guangzhou, Guangdong China

**Keywords:** Recurrent spontaneous abortion (RSA), MYC-associated factor X (MAX), Human endometrial stromal cells (HESCs), Decidualization, Odd-skipped related transcription factor 2 (OSR2)

## Abstract

Human uterine stromal cell undergoes decidualization for pregnancy establishment and maintenance, which involved extensive proliferation and differentiation. Increasing studies have suggested that recurrent spontaneous abortion (RSA) may result from defective endometrial stromal decidualization. However, the critical molecular mechanisms underlying impaired decidualization during RSA are still elusive. By using our recently published single-cell RNA sequencing (scRNA-seq) atlas, we found that MYC-associated factor X (MAX) was significantly downregulated in the stromal cells derived from decidual tissues of women with RSA, followed by verification with immunohistochemistry (IHC) and quantitative real-time polymerase chain reaction (qRT-PCR). MAX knockdown significantly impairs human endometrial stromal cells (HESCs) proliferation as determined by MTS assay and Ki67 immunostaining, and decidualization determined by F-actin, and decidualization markers. RNA-seq together with chromatin immunoprecipitation sequencing (ChIP-seq) and cleavage under targets and release using nuclease sequencing (CUT&RUN-seq) analysis were applied to explore the molecular mechanisms of MAX in regulation of decidualization, followed by dual-luciferase reporter assay to verify that MAX targets to (odd-skipped related transcription factor 2) OSR2 directly. Reduced expression of OSR2 was also confirmed in decidual tissues in women with RSA by IHC and qRT-PCR. OSR2 knockdown also significantly impairs HESCs decidualization. OSR2-overexpression could at least partly rescue the downregulated insulin-like growth factor binding protein 1 (IGFBP1) expression level in response to MAX knockdown. Collectively, MAX deficiency observed in RSA stromal cells not only attenuates HESCs proliferation but also impairs HESCs decidualization by downregulating OSR2 expression at transcriptional level directly.

**Supplementary information:**

The online version contains supplementary material available at 10.1007/s00441-022-03579-z.

## Introduction

Recurrent spontaneous abortion (RSA) is generally defined as two or more pregnancy losses before 24th gestational week, occurs in 1–5% of women during reproductive age, with an increasing incidence year by year (Bender et al. 2018; Liu et al. 2021; Yu et al. 2021). Furthermore, the risk of pregnancy complications, such as preeclampsia as well as fetal growth restriction, was significantly increased in women who had ever experienced RSA (Ali et al. 2020; Cozzolino et al. 2019). Undoubtedly, women suffering from RSA are vulnerable to both physical and psychological stress. To date, our understanding of the etiological causes of RSA still remains far from sufficient, though lots of factors are recognized to result in RSA, such as abnormal chromosome karyotype of parents or embryos, abnormal uterine anatomy, infection, endocrine disorders, metabolic or autoimmune diseases (Krieg and Westphal 2015). About half of the etiological causes are elusive, called unexplained RSA (Liu et al. 2021). Recent studies indicated that unexplained RSA is associated with abnormal expression of certain transcription factors, like signal transducer and activator of transcription 3 (STAT3) and storkhead box 1 (STOX1) in endometrium (Li et al. 2018; Zhu et al. 2021). However, the molecular and cellular mechanisms underlying RSA are incompletely understood (Ali et al. 2020; Muyayalo et al. 2018).

By using single-cell RNA sequencing (scRNA-seq) analysis, recent studies have characterized molecular and cellular atlas and cellular communication patterns elaborately at the placental-decidual interface in human pregnancy (Huang et al. 2021; Nelson et al. 2016; Suryawanshi et al. 2018; Vento-Tormo et al. 2018). And the integral immune-conflicts and defective decidua stromal niche related to RSA were highlighted (Du et al. 2021; Wang et al. 2021). These studies overall illustrated the critical role of the decidua throughout pregnancy. Decidua, with high heterogeneity, is one of the fundamental tissues within the maternal–fetal interface composed of decidua basalis as well as fetal placenta. A variety of cell types in decidual tissues, like decidual stromal cells, epithelial cells, natural killer cells, macrophages, and T cells have been more comprehensively disclosed with recent development of scRNA-seq (Du et al. 2021; Wang et al. 2021).

Increasing studies have suggested that RSA may result from defective endometrial stromal decidualization (Du et al. 2021; Jiang et al. 2020; Larsen et al. 2013). Decidualization is a process, adaptative to pregnancy, that occurs in endometrium of women during the secretory phase of menstrual cycle as well as during pregnancy and is implicated in the differentiation of stromal fibroblasts into a specialized type of cells termed decidual stromal cells (Geisert et al. 2012). To date, the molecular as well as cellular mechanisms of decidualization have been studied for many years, revealing many transcriptional factors implicated in regulation, including progesterone receptor (PR), forkhead box O1 (FOXO1), myeloid ecotropic virus insertion site 1 (MEIS1), and homeobox gene HOXA10 (Mazur et al. 2015; Park et al. 2016; Xu et al. 2020). However, the critical molecular mechanisms underlying impaired decidualization during RSA are still elusive.

We found that MYC-associated factor X (MAX), a transcription factor belonging to basic helix-loop-helix leucine zipper (bHLH-Zip) family, was significantly downregulated in the stromal cells derived from decidual tissues of women with RSA using our recently published scRNA-seq atlas (Du et al. 2021).

MAX can form MAX–MAX homodimers and heterodimers with other family members, including MYC and the MAX dimerization protein (MXD) family (Carroll et al. 2018; Diolaiti et al. 2015; Hurlin and Huang 2006). MYC is a proto-oncogene related to various biological processes, including cell proliferation, differentiation, and apoptosis (Carroll et al. 2018; Hurlin and Huang 2006). The homodimers and heterodimers compete to bind a common DNA sequence motif, called the E box (Augert et al. 2020). It has been reported that MYC and MAX are up-regulated in some endometrial cancer samples (Kandoth et al. 2013). However, so far, the roles of MAX in either endometrial decidualization or RSA have not yet been reported.

We hypothesized that abnormally decreased expression of MAX might contribute to the pathogenesis of RSA by impairing endometrial stromal cells proliferation and decidualization. The purpose of this study was to shed new light on the molecular mechanisms related to impaired decidualization in RSA women by elucidating the roles of MAX in regulation of proliferation and decidualization in human endometrial stromal cells (HESCs) in vitro and decidual tissues in vivo from women with RSA.

## Materials and methods

### Human subjects and tissue collection

Ethical approval for our study was acquired from the Medical Ethics Committee of The Third Affiliated hospital of Guangzhou Medical University (ethics approval number: 20170126). This study was performed following Declaration of Helsinki. All recruited volunteers provided written informed consents. Women with regular menses, those either undergoing elective termination of normal pregnancies without any miscarriage history, or those with RSA were recruited for this study. Women who have taken hormonal therapy in 3 months before the surgery were excluded from this study. Women with abnormal karyotype, abnormal uterine anatomy, infection, endocrine disorders, metabolic, or autoimmune diseases were also excluded. Patients who underwent termination of first trimester pregnancy and with more than once previous spontaneous unexplained miscarriage, were recruited in RSA group. Women who underwent elective termination of a normal early pregnancy without history of miscarriage or any other pregnancy complications were included in the normal group. Characteristics of participants used for scRNA-seq are summarized in Supporting Information of our published literature (Du et al. 2021). Characteristics of participants used for quantitative real-time polymerase chain reaction (qRT-PCR) as well as immunohistochemistry (IHC) are summarized in Supplementary Table S1. Decidual tissues were collected through ultrasound-guided curettage. Decidual tissues were identified macroscopically and washed with phosphate-buffered saline (PBS). Decidual tissues used for qRT-PCR were soon flash-frozen in liquid nitrogen and stored at − 80 ℃ for further use. Decidual tissues for IHC staining were fixed with formalin followed by paraffin-embedded. All decidual sections were evaluated and reviewed by at least two experienced pathologists to confirm the histologic assessment.

### scRNA-seq data reanalysis

Our recently published scRNA-seq data of first trimester decidua from RSA and control decidua tissues was reanalyzed to identify the expression pattern of MAX and odd-skipped related transcription factor 2 (OSR2) (Du et al. 2021). Data quality characteristic of scRNA-seq of 11 samples (5 derived from women with normal pregnancy, and 6 derived from women with RSA) are summarized in Supporting Information of our published literature (Du et al. 2021). Analysis of the data was performed via R package, Seurat (Version 4.0.0). Low-quality cells that expressing less than 500 genes were discarded. After normalizing the data, the clusters were recognized with the expression of markers (Du et al. 2021). Specific gene expression was plotted with Vlnplot function of Seurat by group or cluster, and the average expression in the specific cluster was calculated and plotted as boxplot with R package “ggplot2.”

### Immortalized HESCs culture and decidualization induction

Immortalized HESCs cell line, a telomerase immortalized benign endometrial stromal cell line, was obtained from the American Type Culture Collection (ATCC, USA) (Krikun et al. 2004). HESCs were then cultured as described previously (Liao et al. 2015). Specifically, cells were cultured at 37 °C with 5% CO_2_ in a humidified chamber in DMEM/F12 (Sigma, USA) containing 1 mM sodium pyruvate and 3.1 g/l glucose, supplemented with 10% charcoal-stripped fetal bovine serum (CS-FBS, Biological Industries, Israel), 1% insulin-transferrin-selenium (ITS, Gibco, UK), 500 ng/ml puromycin (Sigma, USA), and 1% penicillin streptomycin (PS, Gibco, UK). As for induction of decidualization, HESCs were cultured in 2% DMEM/F12 media containing 2% CS-FBS as well as 1% PS overnight before treatment with differentiation medium supplemented with 1 mM medroxyprogesterone acetate (MPA, Sigma, USA) as well as 0.5 mM dibutyryl cAMP (db-cAMP, MCE, USA) according to previous report (Brighton et al. 2017). The medium was replaced every 2 days.

### RNA interference

All small-interfering RNA (siRNA) oligonucleotides were obtained from Ribo Biological Technology (Ribo, China). Transfections with MAX siRNA (siMAX) or OSR2 siRNA (siOSR2) in parallel with non-targeting scrambled siRNA (siCON) for negative control were performed in HESCs using a LipofectamineTM RNAiMAX Transfection Reagent (Invitrogen, USA), at about 50% confluence following the manufacturer’s instructions. Transformation of the medium was performed after 6 h, and the medium was replaced with differentiation medium as required. The sequences of the siRNA are listed showed in Supplementary Table S2.

### MTS assays

HESCs viability was determined using the MTS cell proliferation assay (Promega, USA). In Brief, HESCs were subjected into a 96-well plate (Thermo Fisher, USA) with a density of 1.5 × 10^3^ per well. Viable HESCs were detected through optical density (OD) at 490 nm at different time points, viable HESCs were detected. Experiments were repeated for at least three times for each sample.

### Immunostaining

The tissues samples derived from normal early pregnant women requesting pregnancy termination and women with RSA, and tissue samples at different phase of the menstrual cycles were used for immunohistochemistry. Immunohistochemistry analysis was carried out as described previously (Zhang et al. 2014). Briefly, the fixation of the tissues samples was performed using formalin. Then, the samples were embedded in paraffin, followed by sectioned onto glass slides. The following primary antibodies were used: anti-MAX Rabbit polyclonal (1:200, Abcam, UK), anti-OSR2 rabbit polyclonal antibody (1:200, LifeSpan BioSciences, USA), anti-F-actin mouse monoclonal antibody (1:200, Abcam, UK). Moreover, HESCs cultured on glass chamber slides with transfection by scrambled siRNA or MAX-targeting siRNA were subjected to immunofluorescence staining as described (Liao et al. 2015). Briefly, 4% paraformaldehyde (PFA, CST, USA) was utilized to fix HESCs for 5 min at room temperature first. After PBS washing, HESCs underwent permeabilization by using 0.1% Triton X-100 for 10 min followed by blocked in 0.5% bovine serum albumin (BSA, CST, USA) in PBS, at room temperature, for an hour. Subsequently, anti-MAX rabbit polyclonal antibody (1:200, CST, USA) and anti-Ki67 rabbit monoclonal antibody (1:200, Abcam, UK) primary antibodies were used respectively. Fluorescence (cyanine 2 or cyanine 3)-conjugated secondary antibodies (CST, USA) were utilized to show signals. And staining of nuclei was performed by using DAPI (1 μg/ml, Sigma, USA).

### RNA isolation and qRT-PCR

Total RNA was extracted with RNAiso Plus (TaKaRa, Japan) and reverse transcribed with PrimeScript RT Reagent Kit (Takara, Japan). qRT-PCR was used to determine the gene expression by using TBGreen® Premix Ex TaqTM II (TaKaRa, Japan) with QuantStudio 5 Real-Time PCR System (Applied Biosystems, USA). The primer sequences are shown in Supplementary Table S2. The mRNA expression levels were normalized with an average CT value of the three housekeeping genes, including *GAPDH* (glyceraldehyde 3-phosphate dehydrogenase), *ACTB* (actin beta), and *SDHA* (succinate dehydrogenase complex flavoprotein subunit A).

### RNA-seq

With transfection with MAX-targeting siRNA (siMAX) or siCON for negative control, HESCs were cultured in differentiation medium supplemented with MPA and cAMP and collected at day 3 as mentioned above. Total RNA from cell samples was extracted and employed for RNA sequencing (RNA-seq) analysis. Constructions of the cDNA library were carried out followed by single-end sequencing of 50 bp on BGIseq500 platform. Bowtie2 was applied to aligned high-quality reads to the human reference genome (GRCh38), then the gene expression level was calculated according to the method of RNA-Seq by Expectation Maximization (RSEM). Essentially, differential gene expression was performed using the DESeq statistical model. Heatmaps were generated using GraphPad Prism 8 Software (GraphPad Software, USA).

### Databases searching

Gene Expression Omnibus (GEO) DataSet Brower was used to generate the graph of OSR2 expression in endometrium throughout the menstrual cycle (Talbi et al. 2006). And the graph could be downloaded from the following website (https://www.ncbi.nlm.nih.gov/geo/query/acc.cgi?acc =). Transcriptomic data of OSR2 expression in 27 different tissue samples from 95 human individuals were obtained from National Center for Biotechnology Information (NCBI) Gene Expression Database (Fagerberg et al. 2014). And the graph could be downloaded from the following website (https://www.ncbi.nlm.nih.gov/gene/116039). The Integrative Genomics Viewer software (IGV, Broad Institute, USA) was used to generate the graph of PR occupation at the OSR2 promoter compared with input assayed by chromatin immunoprecipitation sequencing (ChIP-seq) during HESCs decidulization. The figures (Supplementary Figs. S3a, b and S4) are used with permissions from the publisher.

### Overexpression vectors and transfection

All plasmids were purchased from MiaoLing Plasmid Sharing Platform (MLPSP, China). All plasmids were confirmed by DNA sequencing. Overexpression efficiency was confirmed through qRT-PCR and WB analysis. The virus was generated by Genepharma Company. An empty pLVX-IRES-ZsGreen vector served as a negative control for MAX or OSR2 overexpression. Thereafter, transfection of lentiviral transfer plasmids was performed by using packaging mix in HEK 293 T cells (ATCC, USA). Seventy-two hours after transfection, the virus was concentrated by ultracentrifugation and used to infect HESCs respectively. Thereafter, HESCs were transfected with siMAX or siCON for 24 h, and cultured in differentiation medium containing MPA as well as cAMP up to 3 days.

### WB analysis

Protein extraction were carried out, followed by WB analysis as described previously (Zhang et al. 2014). Proteins were separated with SDS–polyacrylamide gel electrophoresis (PAGE) as well as transferred onto polyvinylidene fluoride membranes (PVDF, Millipore, USA). The primary antibodies were applied according to the provider’s recommendations: anti-MAX (1:200, CST, USA), anti-IGFBP1 (1:1000, Abcam, UK), anti-β-tubulin (1:5000, Abclonal, China). β-Tubulin was used as a loading control. Immunoblotting analysis was performed with Supersignal West Pico Chemiluminescent substrate (Thermo Fisher, USA).

### ChIP-seq

HESCs were cultured in differentiation medium containing MPA as well as cAMP for 3 days. Subsequently, 1% formaldehyde PBS solution was used to fixed HESCs (1 × 10^7^ cells) for 10 min at room temperature. Then, the termination of cross-linking was performed on ice for 10 min by using 125 mM glycine. Dulbecco’s phosphate buffered saline (DPBS, Gibco, UK) was used to wash HESCs, which were then scraped and centrifuged at 700* g* for 5 min at 4 °C. Thereafter, the supernatant was discarded while the sediment was flash-frozen in liquid nitrogen and transported on dry ice immediately. MAX ChIP was performed by Active Motif (USA) company. The DNA was sheared into smaller fragments by sonication. Chromatin immunoprecipitation was conducted using the anti-MAX (proteintech, China) antibody. MAX-bound DNA was purified and amplified to build sequencing libraries. One hundred fifty base pair paired-end read sequencing reactions were then performed on an Illumina Hi-sequencer. The raw ChIP-seq reads were aligned to the human reference genome (GRCh38) utilizing BWA software. Identification of regions of ChIP-seq enrichment and normalization of ChIP against the input control in an unbiased manner were performed by utilizing model-based analysis of ChIP-Seq (MACS) peak searching algorithm. The bigWig files were displayed on washU browser. ChIP-seq experiment was performed from one biological replicate.

### CUT&RUN-seq

HESCs were cultured in differentiation medium containing MPA and cAMP for 3 days. Subsequently, HESCs were bound to concanavalin A (ConA) beads. Permeabilization was performed with a digitonin containing buffer. Incubation with antibodies against MAX (proteintech, China) was set at 4 °C overnight. MAX-bound DNA was then purified and amplified to build libraries for cleavage under targets and release using nuclease sequencing (CUT&RUN-seq) or be used as templates for CUT&RUN-qRT-PCR. Negative control CUT&RUN assays were carried out with rabbit IgG antibody. One hundred fifty base pair paired-end sequencing were then carried out on the Illumina NovaSeq 6000 instrument by Epibiotek company (China). CutRunTools were employed to facilitate identify chromatin-related protein binding, followed by genomic footprinting analysis as described before (Zhu et al. 2019). In brief, by utilizing Bowtie2, sequences were aligned to the genome assembly. MACS2 was used for peak calling under parameters: “-f BAMPE -q 0.01 -B –SPMR –keep-dup all.” Identified peaks were chosen for motif analysis using MEME. CUT&RUN-seq experiment was performed from one biological replicate. The sequences of the primers used in CUT&RUN-qRT-PCR are listed in Supplementary Table S2.

### Dual-luciferase reporter assay

All plasmids were obtained from MLPSP (China). OSR2 promoter regions, ranging from 2000 bp upstream of the transcriptional start site to 100 bp downstream, amplified from human genomic DNA using PCR, were inserted into upstream of luciferase gene of pGL4-Basic vector, respectively. The sequences of the plasmids were confirmed by plasmid DNA sequencing. For eliminating differences in transfection efficiency or cell number, pGL4.74 (hRluc-TK) encoding Renilla luciferase was employed as an internal reference. HEK293T cells were cultured in DMEM (Hyclone, USA) containing 10% FBS (Hyclone, USA) followed by used for luciferase reporter assay. HEK293T cells were subjected into 24-well plates. Each plate was transiently co-transfected by these two vectors with or without a MAX overexpression vector. Transfection of all plasmids into HEK293T cells was performed by utilizing Lipofectamine™ 3000 reagent (Invitrogen, USA). Assays were performed three biological replicates by utilizing Dual-Lumi™ II Luciferase Reporter Gene Assay Kit (Beyotime Biotechnology, China).

### Statistical analysis

Images were assembled in Photoshop Software (Adobe Systems Version 7.0, CA) and Adobe Illustrator (Adobe Systems Version 19.1.2, CA). SPSS Software (IBM SPSS Statistics Version 21.0, IBM Corp., USA) and GraphPad Prism 8 Software (GraphPad Software, USA) were utilized to perform statistical analysis. All results are expressed as the mean ± SEM from at least 3 independent experiments. Statistical analyses were carried out by utilizing two-tailed Student’s *t*-tests, one-way ANOVA, or two-way ANOVA. Statistical significance was inferred at a two-tailed value of *P* < 0.05.

## Results

### Aberrantly downregulation of decidual MAX is correlated with RSA

**Fig. 1:**
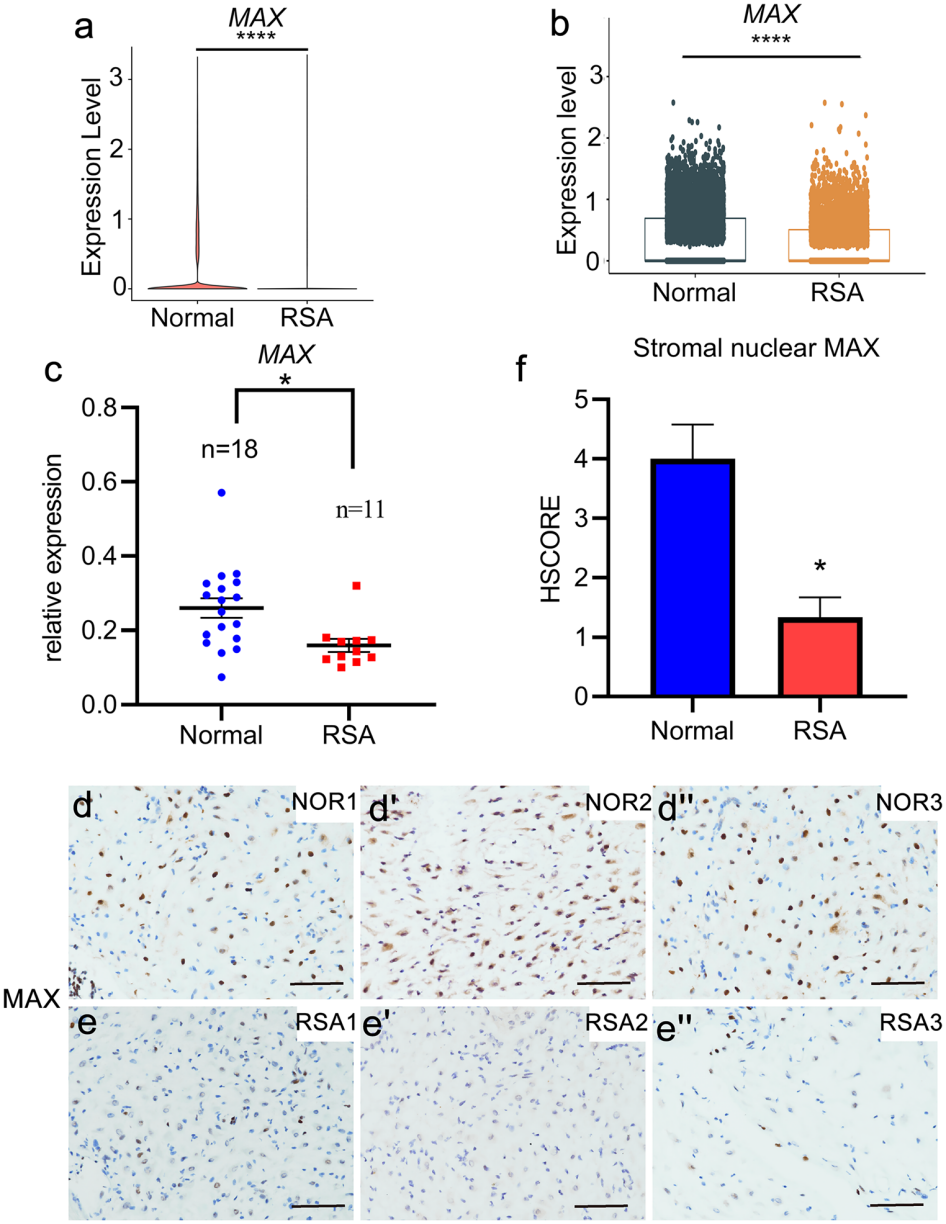
Expression of decidual MAX is abnormally attenuated in women with RSA. (**a**) Single-cell RNA-sequencing (scRNA-seq) analysis of MYC-associated factor X (*MAX*) expression in deciduas derived from women with recurrent spontaneous abortion (RSA) (*n* = 6) and those with normal early pregnancies (*n* = 5) by using VlnPlot functions in single-cell R tool kit Seurat. ****P < 0.0001. (**b**) scRNA-seq analysis of *MAX* expression in decidual stromal cells derived from women with RSA (*n* = 6) and those with normal early pregnancies (*n* = 5) by using boxPlot functions in single cell R tool kit Seurat. ****P < 0.0001. (**c**) *MAX* mRNA level in deciduas derived from 11 women with RSA and 18 women with normal early pregnancies were detected by quantitative real-time polymerase chain reaction (qRT-PCR). CT values were normalized to an average CT value of three housekeeping genes, including *GAPDH* (glyceraldehyde 3-phosphate dehydrogenase), *ACTB* (actin beta) and *SDHA* (succinate dehydrogenase complex flavoprotein subunit A), indicated as the mean ± SEM. ****P < 0.0001. (**d-d''**, **e-e''**, and **f**) Protein level and quantification of MAX in decidual tissue. NOR, normal abortion group; RSA, recurrent spontaneous abortion group; S, stroma. *P < 0.05. Scale, 100 μm

scRNA-seq can characterize transcriptome profiles of specific cell types at a much higher resolution, compared to the traditional RNA-seq. Our recently published scRNA-seq atlas of first trimester decidua from five normal samples as well as six RSA patients (Du et al. 2021) showed that MAX was significantly downregulated in RSA group, indicating its possible role in maintaining normal pregnancy (Fig. 1a). Moreover, MAX was abundantly expressed in different cell types of decidual tissues of women with normal early pregnancies, including decidual stromal cells, peripheral vascular cells, and endothelial cells, among which MAX was mainly expressed in decidual stromal cells (Supplementary Fig. S1). Because MAX is specifically and abundantly expressed in decidual stromal cells, this cell type is the focus of the present study. The single-cell analysis of decidual stromal cells confirmed that MAX was significantly downregulated in RSA group (Fig. 1b). Furthermore, the expression of MAX was further evaluated in the decidual tissues of women with normal pregnancy or RSA. Consistent with the scRNA-seq results, MAX was significantly downregulated in RSA group at mRNA levels as assayed by qRT-PCR (Fig. 1c). IHC analysis showed that MAX expression declined in decidual tissues of women with RSA (Fig. 1d-d', e-e', f**)**. Overall, the above results indicated that aberrantly downregulation of decidual MAX is correlated with RSA.

### MAX is abundantly expressed in human endometrium and decidualized HESCs in vitro

**Fig. 2:**
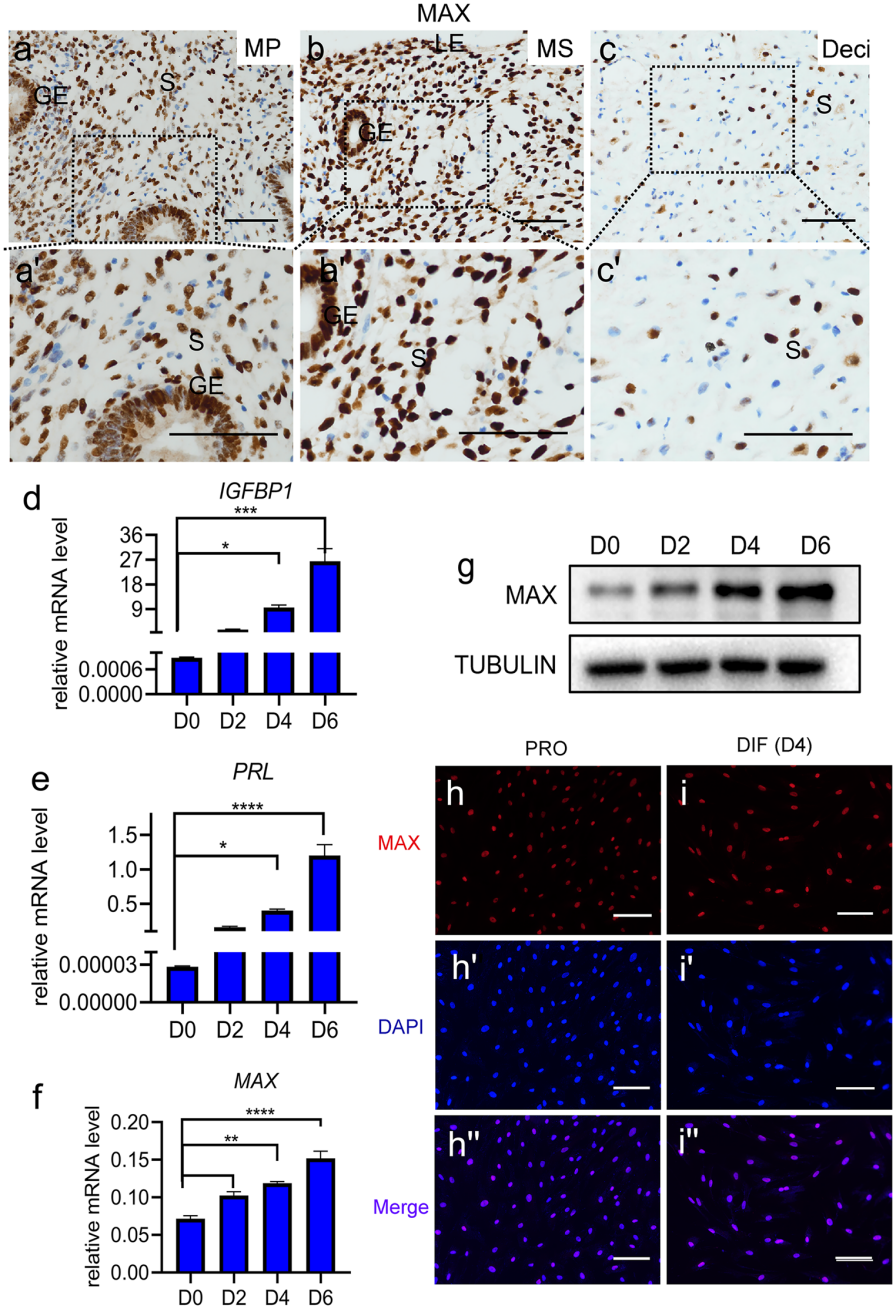
MAX is abundantly expressed in human endometrium in vivo as well as HESCs in vitro. (**a-a'**, **b-b'**, and **c–c'**) MAX expression in human endometrium was detected by immunohistochemistry (IHC) staining. MP, mid-proliferation endometrium; MS, mid-secretory endometrium; Deci, early pregnancy deciduas; S, stroma; GE, glandular epithelium; LE, luminal epithelium. Scale bars,100 μm. *n* = 3. (**d**–**f**) Insulin-like growth factor binding protein 1 (*IGFBP1*), prolactin (*PRL*), and *MAX* mRNA level in decidualized human endometrial stromal cells (HESCs) cultured in differentiation medium for 0–6 days were detected by qRT-PCR. CT values were normalized to were normalized with an average CT value of the three housekeeping genes, including *GAPDH*, *ACTB*, and *SDHA*, indicated as the mean ± SEM (*n* = 3). *P < 0.05; ***P < 0.001; ****P < 0.0001. (**g**) The protein levels of MAX in differentiated HESCs after treatment with medroxyprogesterone acetate (MPA) and dibutyryl cAMP (db-cAMP) for 0, 2, 4, and 6 days were detected by western blot (WB). *n* = 3. TUBULIN was utilized as a loading control. (**h**–**h''** and **i**-**i''**) Immunofluorescence staining of MAX in proliferative or differentiation medium HESCs at day 4. PRO, proliferation; DIF, differentiation. Scale bars, 100 μm

The findings described above encouraged us to further investigate the expression of MAX in the endometrium and HESCs. IHC showed that the MAX protein was abundantly expressed in the mid-proliferative as well as mid-secretary phases of human endometrial tissues and decidual tissues from early pregnancy women (Fig. 2a-a', b-b', c–c'), and was located in stromal cell nuclei (Fig. 2a-a', b-b', c–c'). The abundantly sustained expression as well as nuclear localization of MAX in human endometrium suggested that MAX plays a potential role in the endometrial cyclical remodeling during menstrual cycles. Immortalized HESCs were employed to reveal functions and molecular mechanisms of MAX in the regulation of stromal cell differentiation as described (Liao et al. 2015). Both mRNA and protein levels of MAX increased significantly with the progress of decidualization (Fig. 2d–g). Consistently, MAX was specifically expressed in the nucleus of stromal cells cultured in either proliferation or differentiation medium (Fig. 2h–h'', i-i''). Collectively, the results above demonstrated that MAX may play a significant role in endometrial cyclical proliferation and differentiation.

### MAX is indispensable for normal proliferation of HESCs

**Fig. 3:**
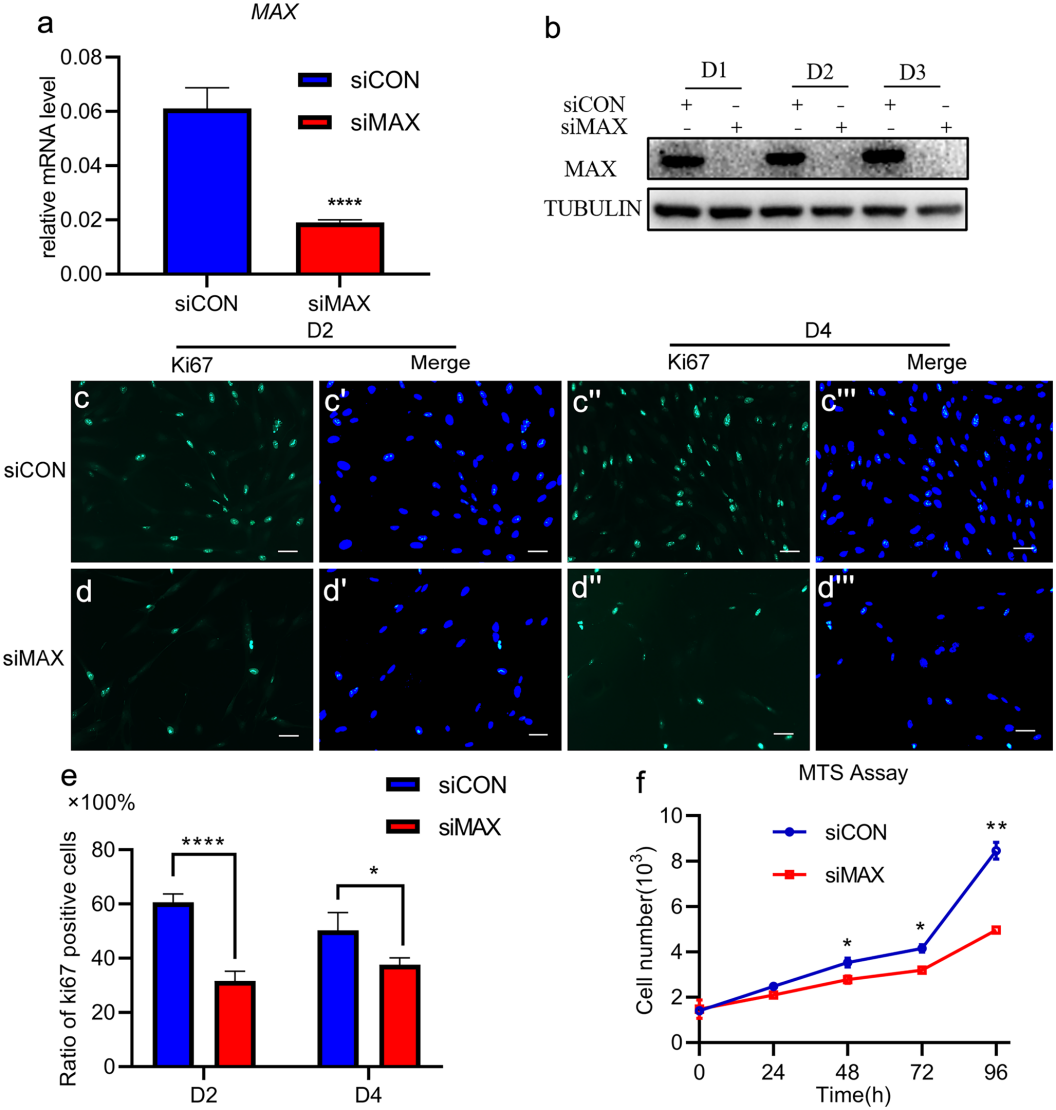
MAX knockdown attenuates HESCs proliferation. (**a**) MAX mRNA level in HESCs cultured in proliferation medium with transfection of scrambled siRNA (siCON) or MAX-targeting siRNA (siMAX) for 48 h. CT values were normalized to were normalized with an average CT value of the three housekeeping genes, including *GAPDH*, *ACTB*, and *SDHA*, indicated as the mean ± SEM (*n* = 3). **P < 0.01. (**b**) WB analysis of the MAX protein levels in HESCs cultured in proliferation medium transfected with siCON or siMAX for 1–3 days. TUBULIN was utilized as a loading control. (**c–c'''**, **d-d'''**, and **e**) Immunofluorescence of Ki67 as well as ratio of Ki67 positive cells in HESCs cultured in proliferation medium with transfection of siCON or siMAX at day 2 and day 4. *n* = 3. *P < 0.05; ****P < 0.0001. Scale bars, 100 μm. (**f**) Cell proliferation measurements using MTS assay in HESCs cultured in proliferation medium transfected with siCON or siMAX for 0–96 h. Mean ± SEM. *P < 0.05; **P < 0.01

As MAX can extensively promote cell proliferation in different cell types (Grandori et al. 2000; Swartling et al. 2014), we proposed the hypothesis that MAX might promote the proliferation of HESCs. To disclose the function of MAX in proliferation of human endometrial stroma, we knocked down *MAX* with siRNA in HESCs. With treatment of MAX-targeting siRNA or scramble siRNA, HESCs were cultured in proliferation medium to verify the knockdown efficacy of *MAX*. As expected, treatment with MAX-targeting siRNA efficiently down-regulated mRNA and protein levels of MAX in proliferative HESCs (Fig. 3a, b). As we expected, percentage of Ki67 positive cells was significantly attenuated with MAX knockdown (Fig. 3c–c''', d-d''', e). Consistently, cell proliferation ability as detected by MTS assay revealed that MAX knockdown could significantly decrease proliferation activity of HESCs (Fig. 3f). Collectively, the results above demonstrated that MAX is indispensable for proliferation of HESCs in vitro.

### Deficiency of MAX impairs HESCs decidualization

**Fig. 4:**
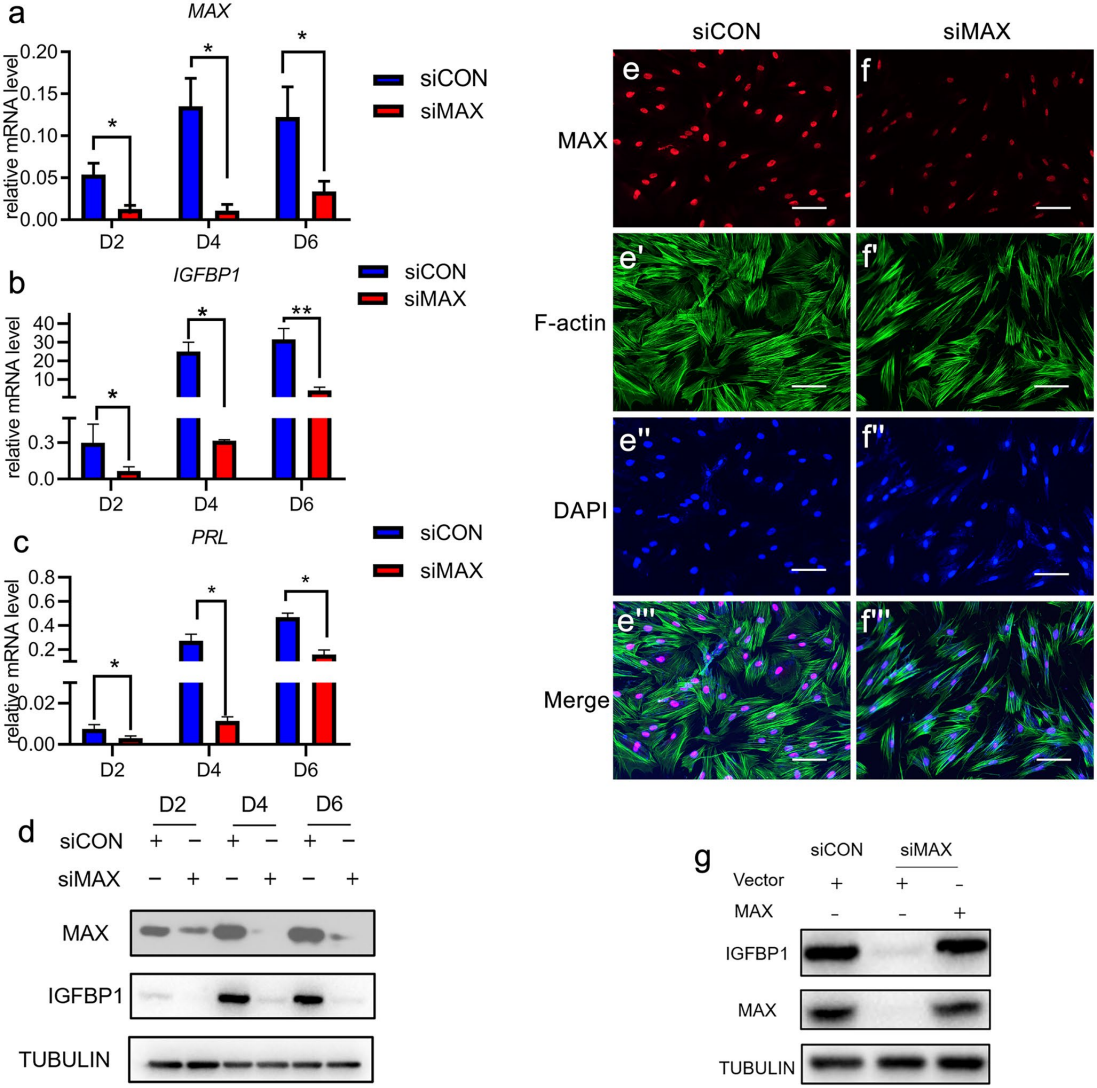
MAX knockdown impaired HESCs decidualization. (**a**–**c**) mRNA level of *MAX*, *IGFBP1* and *PRL* in HESCs transfected with siCON or siMAX followed by cultured in differentiation medium for 2–6 days. CT values were normalized to were normalized with an average CT value of the three housekeeping genes, including *GAPDH*, *ACTB*, and *SDHA*, indicated as the mean ± SEM (*n* = 3). *P < 0.05; **P < 0.01. (**d**) WB analysis of MAX and IGFBP1 in HESCs transfected with siCON or siMAX and cultured in differential culture medium, followed by collection at day 2 to day 6. TUBULIN was used as a loading control. *n* = 3. (**e-e'''** and **f-f'''**) Immunofluorescence shows the actin filaments in HESCs cultured in differentiation medium for 4 days after transfected with siCON or siMAX. Scale bars, 100 μm. *n* = 3. (**g**) WB analysis of IGFBP1 and MAX in HESCs infected with control lentivirus or MAX-overexpression lentivirus for 24 h, and transfection with siCON or siMAX for 24 h, followed by cultured in medium supplemented with MP and cAMP for 3 days. TUBULIN was utilized as a loading control. *n* = 3

As shown in Fig. 2b-b', c–c', MAX was strongly expressed in human endometrium of secretory phase and decidual tissues of early pregnancy. Since endometrial decidualization occurs in women during secretory phase and during pregnancy, knockdown experiments using siRNA were carried out to further ascertain whether MAX play a role during decidualization. Sure enough, both mRNA and protein levels of MAX during decidualization was efficiently reduced by MAX-targeting siRNA (Fig. 4a, d). Furthermore, the mRNA expression levels of two classical decidualization markers, insulin-like growth factor binding protein 1 (IGFBP1) and prolactin (PRL) were significantly attenuated with MAX knockdown (Fig. 4b, c). Consistently, protein level of IGFBP1 was significantly reduced with MAX knockdown during HESCs decidualization (Fig. 4d). The decidualized HESCs undergo dramatically morphological changes with characteristics of by rounded, large, secretory decidual cells, with complex cytoskeletal rearrangements after treatment with differentiation medium containing cAMP and progesterone (Gellersen and Brosens 2014; Strowitzki et al. 2006). The actin filaments in HESCs cultured in differentiation medium were detected by immunofluorescence, and it showed that the normal F-actin polymerization and stress fiber formation was disrupted with decreased elongated stress fibers with MAX knockdown (Fig. 4e-e''', f-f'''), which further confirmed the compromised decidualization. The specificity of the MAX-targeting siRNA target sequences was confirmed by rescue experiments. As expected, infection with MAX-overexpression siRNA resistance lentivirus rescued the decreased IGFBP1 level on MAX knockdown (Fig. 4g). Taken together, the results above suggested that MAX is indispensable for HESCs decidualization.

### MAX directly targets OSR2 in decidualized HESCs

**Fig. 5:**
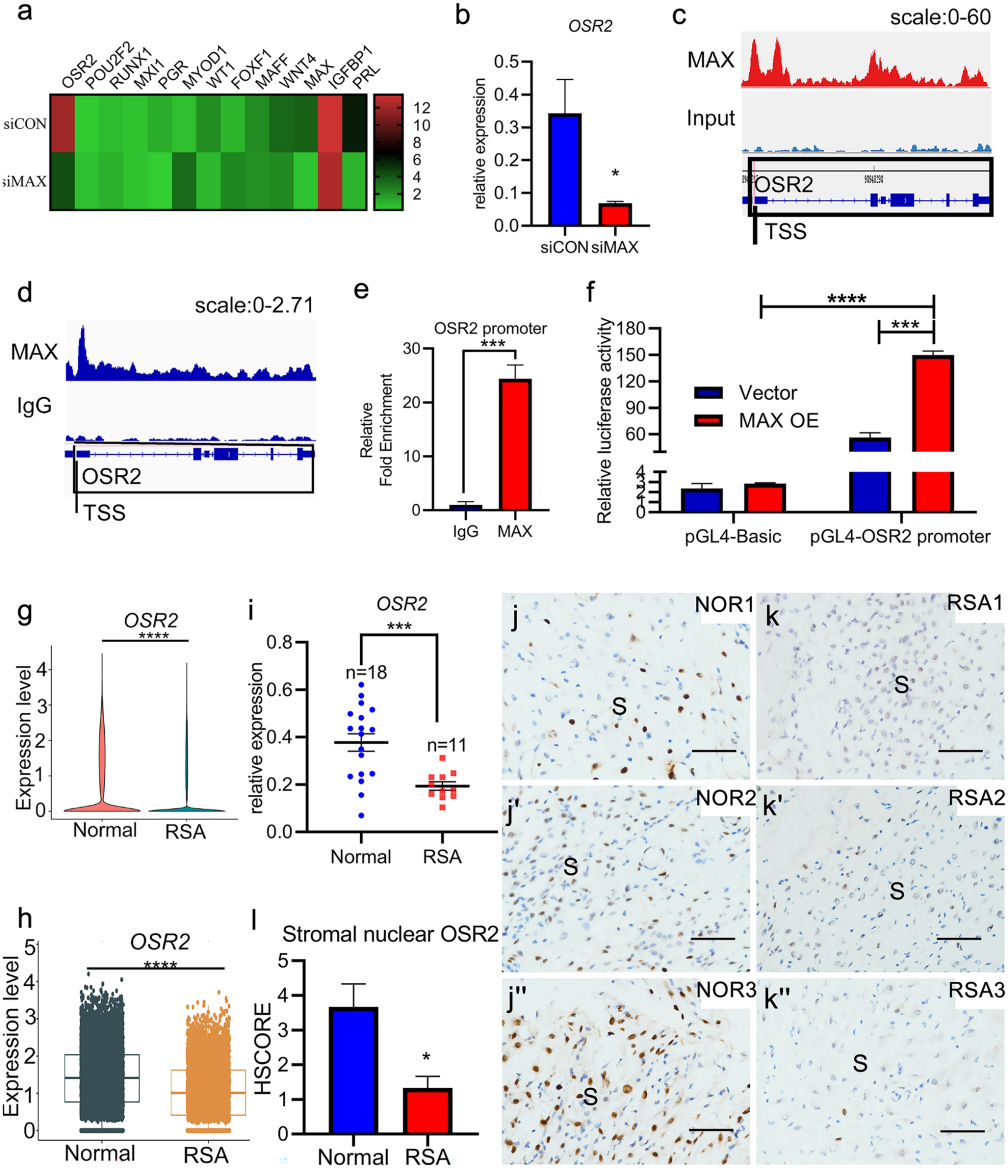
In decidualized HESCs, MAX directly targets to OSR2, of which expression level was decreased in decidual tissues in women with RSA. (**a**) Relative mRNA expression of the most differentially expressed transcription factors, as well as *IGFBP1* and *PRL* in HESCs cultured in differentiation medium for 3 days after transfected with siCON or siMAX were determined by RNA-seq, which were visualized as a heatmap. *n* = 3. (**b**) Relative mRNA expression of odd-skipped related transcription factor 2 (*OSR2*) in HESCs cultured in differentiation medium for 3 days after transfected with siCON or siMAX were determined by qRT-PCR. CT values were normalized to were normalized with an average CT value of the three housekeeping genes, including *GAPDH*, *ACTB*, and *SDHA*, indicated as the mean ± SEM (*n* = 3). *P < 0.05. (**c**) Representative peaks for binding of MAX at the OSR2 promoter compared with input were assayed by Chromatin immunoprecipitation sequencing (ChIP-seq). TSS: transcription start site. (**d** and **e**) Relative enrichment fold and representative peaks for binding of MAX at the OSR2 promoter compared with IgG were assayed by cleavage under targets and release using nuclease qRT-PCR (CUT&CUN-qRT-PCR) and cleavage under targets and release using nuclease sequencing (CUT&RUN-seq), respectively. The occupation of MAX relative to IgG on the promoter on OSR2 is normalized by the occupation of MAX relative to IgG on vacuolar protein sorting 13 homolog B (VPS13B), which is predicted and confirmed as negative occupation according to the database. Values represent the mean ± SEM. *n* = 3. ***P < 0.001. (**f**) Luciferase assays of cis-activation potential of the region containing PGL4-OSR2 promoter or PGL4-Basic, in the presence of MAX over-expression plasmid (MAX OE) comparing with the negative control plasmid (Vector). Values represent the mean ± SEM. *n* = 3. ***P < 0.001; ****P < 0.0001. (**g**) scRNA-seq analysis of *OSR2* expression in deciduas derived from women with RSA (*n* = 6) and those with normal early pregnancies (*n* = 5) by using VlnPlot functions in single-cell R tool kit Seurat. ****P < 0.0001. (**h**) scRNA-seq analysis of *OSR2* expression in decidual stromal cells of women with RSA (*n* = 6) and those with normal early pregnancies (*n* = 5) by using boxPlot functions in single-cell R tool kit Seurat. ****P < 0.0001. (**i**) Relative expressions of *OSR2* in the deciduas of 11 women with RSA and 18 women with normal early pregnancies were detected through qRT-PCR, respectively. CT values were normalized to were normalized with an average CT value of the three housekeeping genes, including *GAPDH*, *ACTB*, and *SDHA*, indicated as the mean ± SEM (*n* = 3). *P < 0.05; ***P < 0.001. (**j-j''**, **k-k''**, and **l**) Immunohistochemical staining shows OSR2 in the deciduas of women with RSA and those with normal early pregnancies and immunostaining results were scored utilizing HSCORE. NOR, normal abortion group; RSA, recurrent spontaneous abortion group; S, stroma. **P < 0.01 Scale, 100 μm

To further investigate the molecular mechanisms by which MAX mediates the regulation of HESCs decidualization, we performed an RNA-Seq analysis on HESCs transfected with siCON or siMAX followed by cultured in differentiation medium for three days. *MAX* mRNA levels and 3 decidualization markers, IGFBP1, PRL, and PR, were reduced significantly with MAX knockdown (Fig. 5a), which were further confirmed by qRT-PCR (Supplementary Fig. S2). The observation was consistent with the results above (Fig. 4b–d).

Because a wide range of transcription factors, such as PR, FOXO1, MEIS1, and HOXA10, have been successfully proved to play critical roles in the regulation of HESCs decidualization (Mazur et al. 2015; Park et al. 2016; Xu et al. 2020), we focused our attention on the most differentially expressed transcription factors. In particular, *OSR2* mRNA level was most significantly decreased with MAX knockdown (Fig. 5a) and the RNA-seq result was further confirmed in independent samples by qRT-PCR (Fig. 5b). In order to clarify the direct target of MAX among the different expressed genes during HESCs decidualization, we performed ChIP-seq experiments by utilizing decidualized HESCs simultaneously. Remarkably, we verified that OSR2 is indeed a direct targeting gene of MAX (Fig. 5c). Likewise, CUT&RUN-seq and CUT&RUN-qRT-PCR further verified the occupancy of MAX in OSR2 promoter during HESCs decidualization (Fig. 5d, e). We performed dual-luciferase assays by using reporter constructs with or without OSR2 promoter in HEK 293 T cell line to further confirm the regulation of the OSR2 expression by MAX. Consistently, the luciferase activity was significantly increased with MAX overexpression (Fig. 5f). These results suggested that MAX directly targets to OSR2 in decidualized HESCs.

Since MAX is significantly down-regulated in RSA as described above, the expression levels of OSR2 detected by scRNA-seq between normal pregnancy group and RSA group were also analyzed. As expected, OSR2 was significantly downregulated in RSA group, both at the overall level and the decidual stromal cells specific level (Fig. 5g, h). Consistently, we observed that the decidual OSR2 expression levels, both mRNA levels and protein levels, were significantly reduced in RSA group than those of the normal pregnancy group (Fig. 5j-j'', k-k'', l). Collectively, these results demonstrated that downregulation of decidual OSR2 is correlated with RSA.

Overall, MAX directly targets to OSR2 in decidualized HESCs. It is likely that MAX deficiency in endometrial stromal cells directly contributes to the down-regulation of OSR2 in endometrial stromal cells, leading to RSA.

### OSR2 is indispensable for HESCs decidualization

**Fig. 6:**
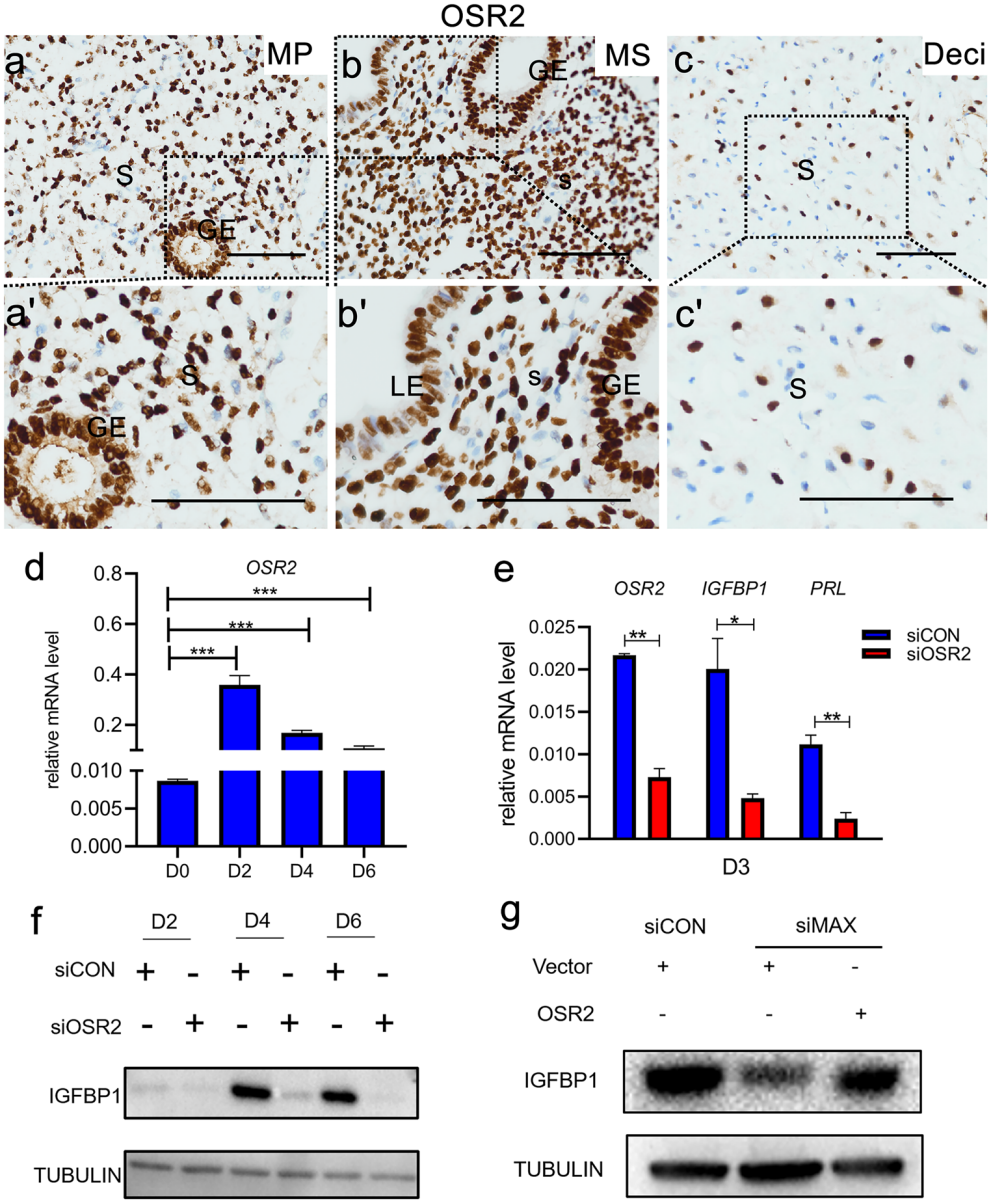
OSR2 is indispensable for HESCs decidualization. (**a-a'**, **b-b'**, and **c–c'**) OSR2 expression in endometrium at different phases and decidual tissues from early pregnancy women was determined by IHC staining. MP, mid-proliferative endometria; MS, mid-secretory endometria; Deci, early pregnancy deciduas; S, stroma; GE, glandular epithelium; LE, luminal epithelium. Scale bars, 100 μm. (**d**) *OSR2* mRNA level in differentiated HESCs cultured in differentiation medium for 0–6 days were detected by qRT-PCR. CT values were normalized to were normalized with an average CT value of the three housekeeping genes, including *GAPDH*, *ACTB*, and *SDHA*, indicated as the mean ± SEM (*n* = 3). *P < 0.05. (**e**) Relative mRNA expression of *OSR2*, *IGFBP1*, and *PRL* in HESCs cultured in differentiation medium for 3 days after transfected with siCON or siOSR2 were determined by qRT-PCR. The values represent the mean ± SEM. *n* = 3. *P < 0.05. **P < 0.01. (**f**) WB analysis of IGFBP1 in HESCs with transfection of siCON or siOSR2 followed by cultured in differential culture medium for 2–6 days. TUBULIN was utilized as a loading control. (**g**) Protein levels of IGFBP1 in HESCs, which were infected with OSR2-overexpression lentivirus or controls for 24 h, followed with transfection of siCON or siMAX for 24 h and finally cultured in differentiation medium for 3 days, were determined by western blotting

To further explored the role of OSR2 in endometrium and decidualization, database searching and our scRNA-seq reanalysis were performed. Excitingly, as shown in Supplementary Fig. S3a, b (Talbi et al. 2006; Fagerberg et al. 2014), we found that OSR2 was specifically and abundantly expressed in endometrium, especially in the mid-secretory phase. Furthermore, OSR2 was mainly expressed in decidual stromal cells (Supplementary Fig. S3c). As shown in Fig. 6a-a', b-b', c–c', the abundantly sustained expression as well as nuclear localization of OSR2 in human endometrium suggested that OSR2 also plays a potential role in the endometrial cyclical remodeling during menstrual cycles. Consistent with the observation, *OSR2* mRNA could be abundantly detected during the in vitro induced decidualization (Fig. 6d). Excitingly, several lines of evidence suggest that OSR2, a transcription factor belonging to C2H2 zinc finger family, may participated in regulation of HESCs decidualization (Cloke et al. 2008; Mazur et al. 2015; Rytkonen et al. 2019) by showing the data from RNA-seq. Nevertheless, none of the studies have provided experimental evidence to prove the hypothesis. To explore the role of OSR2 in decidualization, knockdown experiments were performed by using siRNA. As expected, *OSR2* mRNA was efficiently reduced by OSR2-targeting siRNA on day 3 of in vitro induced decidualization and the mRNA expression levels of two classical decidualization markers, IGFBP1 and PRL, were significantly attenuated with OSR2 knockdown (Fig. 6e). Consistently, IGFBP1 protein level was remarkably reduced with OSR2 knockdown during HESCs decidualization (Fig. 6f). Rescue experiments were performed to further confirm whether OSR2 is indeed regulated by MAX. As expected, infection with OSR2-overexpression lentivirus, rather than control virus, at least partly rescued the downregulated IGFBP1 expression level upon MAX knockdown (Fig. 6g).

Collectively, the results above indicated that MAX regulated OSR2 directly and contributed to the maintenance of decidualization.

## Discussion

RSA is a common pregnancy complication, and its etiology remains largely elusive (Ali et al. 2020; Cozzolino et al. 2019). Notably, accumulating studies have demonstrated that decidualization deficiency is one of the leading causes of RSA (Jiang et al. 2020; Larsen et al. 2013). Normal endometrial decidualization is a prerequisite for successful implantation as well as play critical roles in maintaining pregnancy (Guo et al. 2016; Jiang et al. 2016). So far, the lack of early diagnosis and intervention strategies of RSA makes it difficult to reduce the risk of recurrent abortion in women of childbearing age (Seshadri and Sunkara 2014). We attempted to disclose the potential molecular mechanisms related to RSA in this study, which may result in a novel insight of the pathogenesis of RSA.

**Fig. 7:**
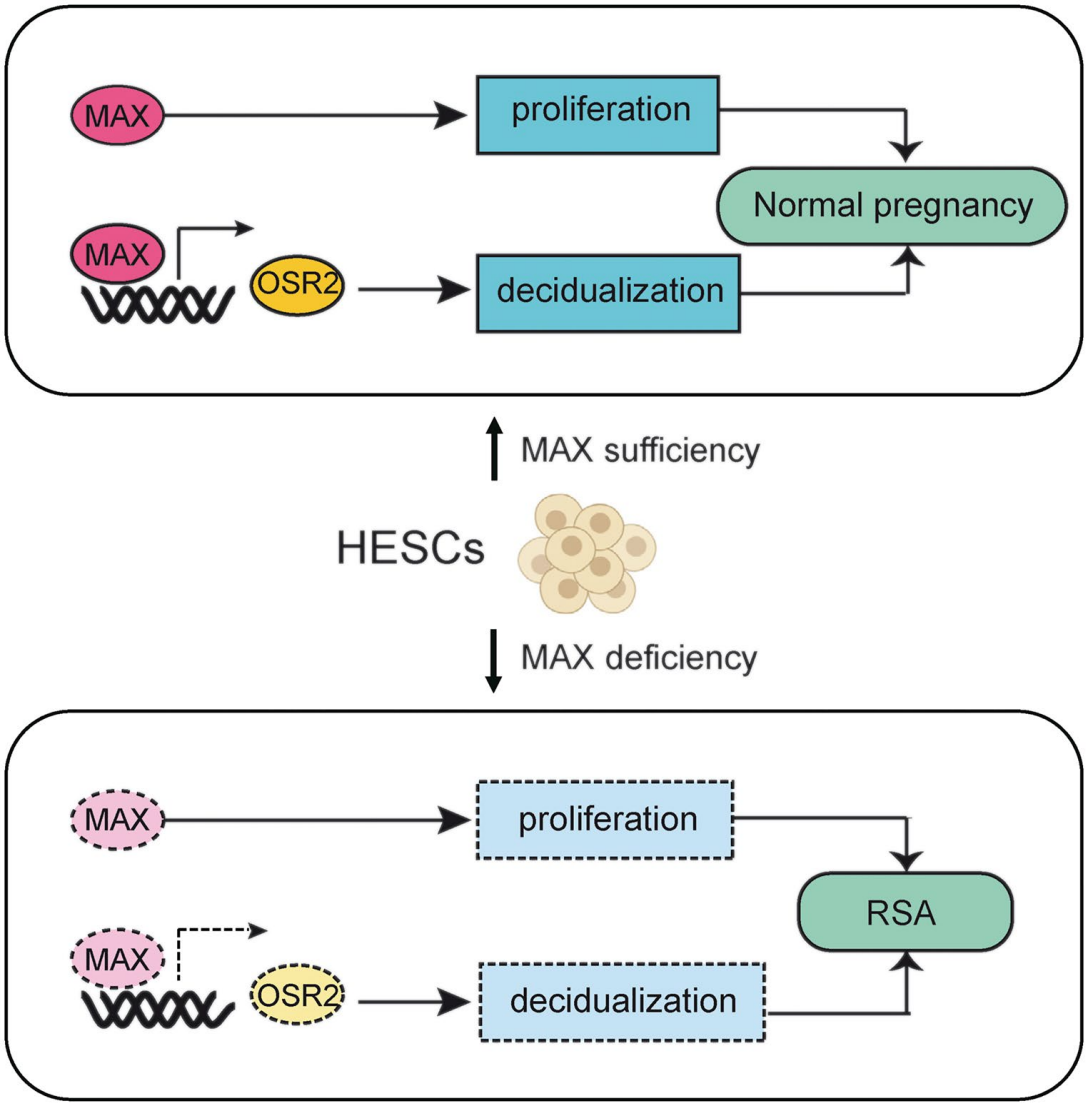
Schematic illustration of the mechanism of the roles of MAX in endometrium of women, promoting proliferation and transcriptionally activating OSR2 to ensure normal decidualization in HESCs, in normal pregnancy. When MAX is sufficient, HESCs proliferate normally and transcriptionally activate OSR2 to ensure normal decidualization, contributing to normal pregnancy, whereas when MAX is deficient, HESCs fail to proliferate normally and are unable to transcriptionally activate OSR2 during decidualization, leading to RSA

By reanalyzing our recent published scRNA-seq data, we found that MAX was significantly downregulated in RSA group both at the overall level and the decidual stromal cell specific level. Decreased MAX expression attenuated the endometrial stromal cells viability and diminished the ratio of Ki67-positive cells. Decreased MAX expression reduced the expression of IGFBP1 and PRL, accompanied with disturbance of cytoskeletal formation during decidualization. We further demonstrated that MAX can directly bind to and transcriptionally activate OSR2. Declined expression of MAX, therefore, reduced OSR2 expression, and suppressed decidualization. A schematic illustration is shown in Fig. 7.

MAX can form MAX-MAX homodimers and heterodimers with MYC or MXD (Carroll et al. 2018). Additionally, MAX serves as a network core between the MYC family members and the MXD family members (Carroll et al. 2018). The essential roles of the MYC family members in promoting proliferation and cell cycle has been well-recognized. MYC-MAX heterodimers can directly bind to genomic E-boxes, recruiting co-activators to enhance active transcription, whereas MXD proteins act as transcriptional repressors, recruiting co-repressors (Carroll et al. 2018). Presumably, the ratio of MYC to MXD is a critical determinant in determining cellular proliferation or differentiation of certain cells (Ayer and Eisenman 1993). Interestingly, MAX and MXD play potential roles in the transformation and differentiation between different cell types, such as adipocyte differentiation and granulosa cell luteinization (Chaffin et al. 2003; Pulverer et al. 2000; Reichert and Eick 1999). Consistently, this study demonstrated that MAX plays an essential role in regulating endometrial stromal cells differentiation. Compared with previous studies, the present study specifically pursued the direct mechanism of MAX in regulation endometrial stromal cells decidualization.

As one of the earliest mesodermal markers, the research on OSR2 is still at its infant (Lan et al. 2001; Stricker et al. 2012). OSR1 is an important paralog of OSR2. It has been indicated that both Osr1 and Osr2 are involved in the regulation of the mesenchymal cell-type differentiation in chicken embryo (Stricker et al. 2012), reminiscent of the differentiation of stromal fibroblasts into decidual cells. In mouse fibroblast mesenchymal cells, Osr2 gene expression is found to be regulated by transcription factors like CCAAT/enhancer-binding protein β (C/EBPβ) as well as transforming growth factor-beta (TGF-β), both of which particulate in regulation of HESCs decidualization (Kawai and Amano 2012; Wang et al. 2012).

Several RNA-seq studies have shown that OSR2 was included in the most significantly down-regulated genes with PR knockdown in endometrial stromal cells during decidualization (Cloke et al. 2008; Mazur et al. 2015), indicating that OSR2 may be regulated by progesterone and participate in regulation of endometrial stromal cells decidualization. Furthermore, as shown in Supplementary Fig. S4, there was PR occupation near the transcription start site (TSS) of the OSR2 promoter during HESCs decidualization (Kaya et al. 2015). Therefore, OSR2 may be regulated by PR directly. Interestingly, the expression patterns of OSR2 were consistent with those of transcription factors essential for endometrial stromal cells decidualization, including PR, HOXA10, MEIS1, HAND2, and promyelocytic leukemia zinc finger (PLZF, also known as ZBTB16) (Gellersen and Brosens 2014; Murata et al. 2020; Szwarc et al. 2018; Xu et al. 2020). Briefly, the expression level of OSR2 increased accompanied with endometrial stromal cells decidualization, and the expression level on day 3 in HESCs induced decidualization in vitro was significantly higher than that on day 8 (Rytkonen et al. 2019), of which tendency approximately consistent with our results. Furthermore, for the first time, we provided experimental evidence to suggest that OSR2 is indispensable for HESCs decidualization.

Interestingly, scRNA-seq analysis of transcriptome characteristics in different phases of the menstrual cycle demonstrated that OSR2 was abundantly expressed during window of implantation (WOI), a narrow window of receptive state well-suited for embryo implantation in both biochemistry and structure (Wang et al. 2020), in stromal fibroblasts, see Extended Data Fig. 6 in Wang et al. (2020). The finding indicated that OSR2 play a potential role in embryo implantation and may contributes to normal pregnancy. Therefore, it is necessary to further investigate how OSR2 participates in regulation of HESCs decidualization in the future.

There are several strengths of our study. Firstly, to our knowledge, it is the first time to reveal the key roles of MAX and OSR2 in endometrial decidualization implicated in RSA. Secondly, the present study uncovered that MAX transcriptionally activates OSR2 by directly binding to its promoter region, to mediate the regulation of HESCs decidualization.

In spite of the strengths, there are some weaknesses that should be acknowledged. Firstly, because we set strict inclusion criteria and focused on unexplained RSA only, the sample number of women’s decidual tissues to verify the relationship of MAX and OSR2 in RSA were relatively small. Thus, it is necessary to strengthen our conclusions on a larger sample scale. Secondly, since the effective commercial OSR2 antibodies for WB and fluorescent immunostaining were not available, we could not show the effect of OSR2 knockdown at protein level and show the colocalization of OSR2 and MAX in the biopsies to provide another verification. Finally, the experimental results are based almost completely on the HESCs cell line. Future research is required to powerfully prove our conclusions by using appropriate animal models.

In conclusion, MAX deficiency observed in RSA stromal cells not only attenuates HESCs proliferation but also impairs HESCs decidualization by downregulating OSR2 expression at transcriptional level directly. Therefore, MAX-OSR2 interaction can be a potential target in early diagnosis and intervention of unexplained RSA caused by impaired decidualization.

## Supplementary Information

Below is the link to the electronic supplementary material.

Supplementary file1 (PDF 95 KB)

Supplementary file2 (DOCX 755 KB)
