# Supplementary material for: MAX deficiency impairs human endometrial decidualization through down-regulating OSR2 in women with recurrent spontaneous abortion
Source: Cell Tissue Res. 2022 Feb 11;388(2):453–69. doi: 10.1007/s00441-022-03579-z (PMC9035420; doi:10.1007/s00441-022-03579-z)
Supplement: Supplementary file 1 — Supplementary file1 (PDF 95 KB) [file 441_2022_3579_MOESM1_ESM.pdf]

Supplementary Table S1. Characteristics of participants used for quantitative real-time polymerase chain reaction (qRT-PCR) and immunohistochemistry.						
	**Age (Years)**	**G/P[c]**	**Ethnicity**	**Gestational weeks**	**Previous unexplained pregnancy loss**	**Embryo chromosome analysis**
**RSA[a]**	34	3/0	HAN/CHINESE	6^{+4}	3	normal
	39	3/1	HAN/CHINESE	6^{+6}	2	normal
	30	5/0	HAN/CHINESE	8^{+2}	4	normal
	31	6/1	HAN/CHINESE	9^{+1}	5	normal
	31	5/0	HAN/CHINESE	8^{+1}	2	normal
	31	11/0	HAN/CHINESE	7^{+3}	11	normal
	37	3/1	HAN/CHINESE	6^{+3}	2	normal
	39	3/0	HAN/CHINESE	6^{+4}	2	normal
	33	3/0	HAN/CHINESE	6^{+4}	3	normal
	28	2/0	HAN/CHINESE	5^{+3}	2	normal
	34	2/0	HAN/CHINESE	7^{+1}	2	normal
**NOR[b]**	33	4/2	HAN/CHINESE	6^{+6}	1	normal
	26	5/1	HAN/CHINESE	6^{+2}	0	normal
	34	3/1	HAN/CHINESE	8^{+5}	0	normal
	32	2/1	HAN/CHINESE	7^{+2}	0	normal
	29	4/3	HAN/CHINESE	6^{+1}	0	normal
	27	3/2	HAN/CHINESE	6^{+6}	0	normal
	21	2/0	HAN/CHINESE	7^{+4}	0	normal
	33	5/2	HAN/CHINESE	6^{+5}	1	normal
	33	4/1	HAN/CHINESE	6^{+3}	1	normal
	33	6/3	HAN/CHINESE	8^{+3}	0	normal
	29	3/1	HAN/CHINESE	9	0	normal
	31	4/2	HAN/CHINESE	6^{+5}	0	normal
	29	5/0	HAN/CHINESE	9^{+2}	0	normal
	31	6/3	HAN/CHINESE	7^{+4}	0	normal
	30	4/1	HAN/CHINESE	6^{+2}	0	normal
	21	1/0	HAN/CHINESE	7^{+3}	0	normal
	28	1/0	HAN/CHINESE	7^{+5}	0	normal
	31	2/0	HAN/CHINESE	9^{+1}	0	normal

[a], recurrent spontaneous abortion; [b], normal pregnancy; [c], gravidity/ parity.

Supplementary Table S2 Primer sequences for qRT-PCR and siRNA sequences (all given from 5′to 3′)

qRT-PCR primer

**Abbreviation**	**Gene**	**Forward primer**	**Reverse primer**
MAX	MYC associated factor X	CGTAGGGACCACATCAAAGAC	TCTGTGGCTTTGTCTAGGATTTG
PRL	Prolactin	CTACATCCATAACCTCTCCTCAG	GGGCTTGCTCCTTGTCTTC
IGFBP1	Insulin-like growth factor binding protein 1	AGAGTCGTAGAGAGTTTAGC	ACACTGTCTGCTGTGATAA
OSR2	odd-skipped related transcription factor 2	TTGACTCCGGACAGAAAGCC	CTGCCGCAAAACTTGCAGAT
GAPDH	Glyceraldehyde-3-phosphate dehydrogenase	ATTTGGCTACAGCAACAGG	TTGAGCACAGGGTACTTTATT

CUT&RUN-qRT-PCR primer

**Abbreviation**	**Gene**	**Forward primers**	**Reverse primers**
OSR2	odd-skippedrelated transcription factor 2	GGGCCGCTACTAAAGCCC	AACCTCTCGCTGTCGACTC
VPS13B	vacuolar protein sorting 13 homolog B	ATGTGAGAAGCAGTGTTGGCT	GGCTGGCTGAAAATGGACAC

siRNA sequences

**Gene**	
siMAX	ACATCGAGGTGGAGAGCGA
siOSR2	GGGCCTCAGTAAATTGACT
