# Supplementary material for: MAX deficiency impairs human endometrial decidualization through down-regulating OSR2 in women with recurrent spontaneous abortion
Source: Cell Tissue Res. 2022 Feb 11;388(2):453–69. doi: 10.1007/s00441-022-03579-z (PMC9035420; doi:10.1007/s00441-022-03579-z)
Supplement: Supplementary file 2 — Supplementary file2 (DOCX 755 KB) [file 441_2022_3579_MOESM2_ESM.docx]

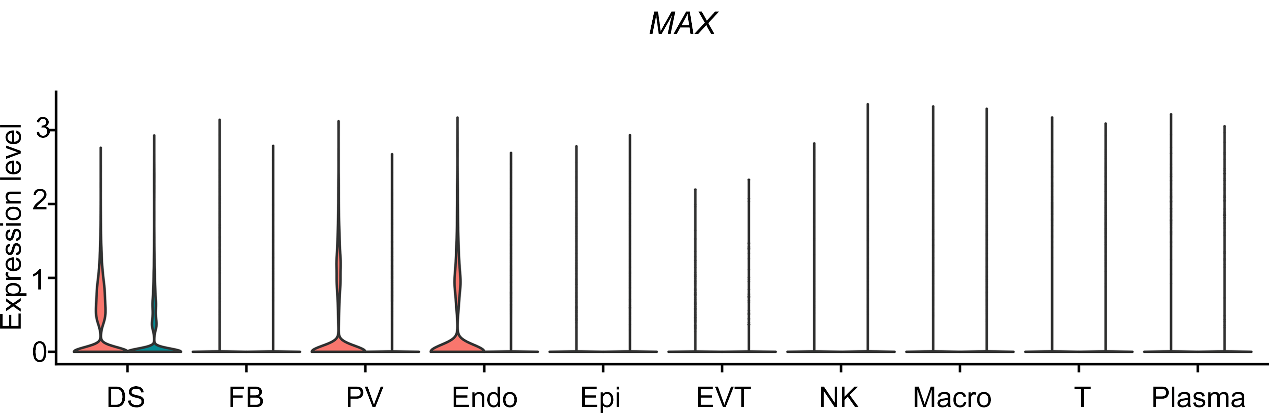


**Supplementary Fig S1.** single-cell RNA-sequencing (scRNA-seq) analysis of MYC associated factor X (MAX) expression in different cell types of deciduas derived from women with recurrent spontaneous abortion (RSA) (n=6) as well as those with normal early pregnancies (n=5) by using VlnPlot functions in single cell R tool kit Seurat. DS: decidual stromal cells; FB: fibroblasts; PV: peripheral vascular cells; Endo: endothelial cells; Epi: epithelial cells; EVT: extravillous trophoblast cells; NK: natural killer cells; Macro: macrophages; T: T cells; Plasma: plasma cells.


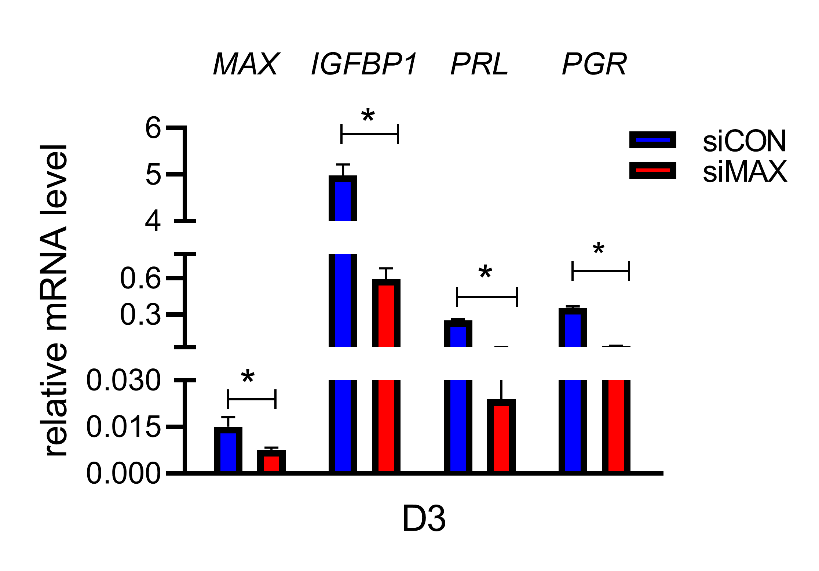


**Supplementary Fig S2.** Relative mRNA expression of *MAX*, insulin-like growth factor binding protein 1 (*IGFBP1*), prolactin (*PRL*), and progesterone receptor (*PGR*) in human endometrial stromal cells (HESCs) cultured in differentiation medium for 3 days after transfected with siCON or siMAX were determined by quantitative real-time polymerase chain reaction (qRT-PCR). CT Values were normalized to an average CT value of three housekeeping genes, including *GAPDH* (glyceraldehyde 3-phosphate dehydrogenase), *ACTB* (actin beta) and *SDHA* (succinate dehydrogenase complex flavoprotein subunit A). Values represent the mean ± SEM. n = 3. **P* < 0.05.


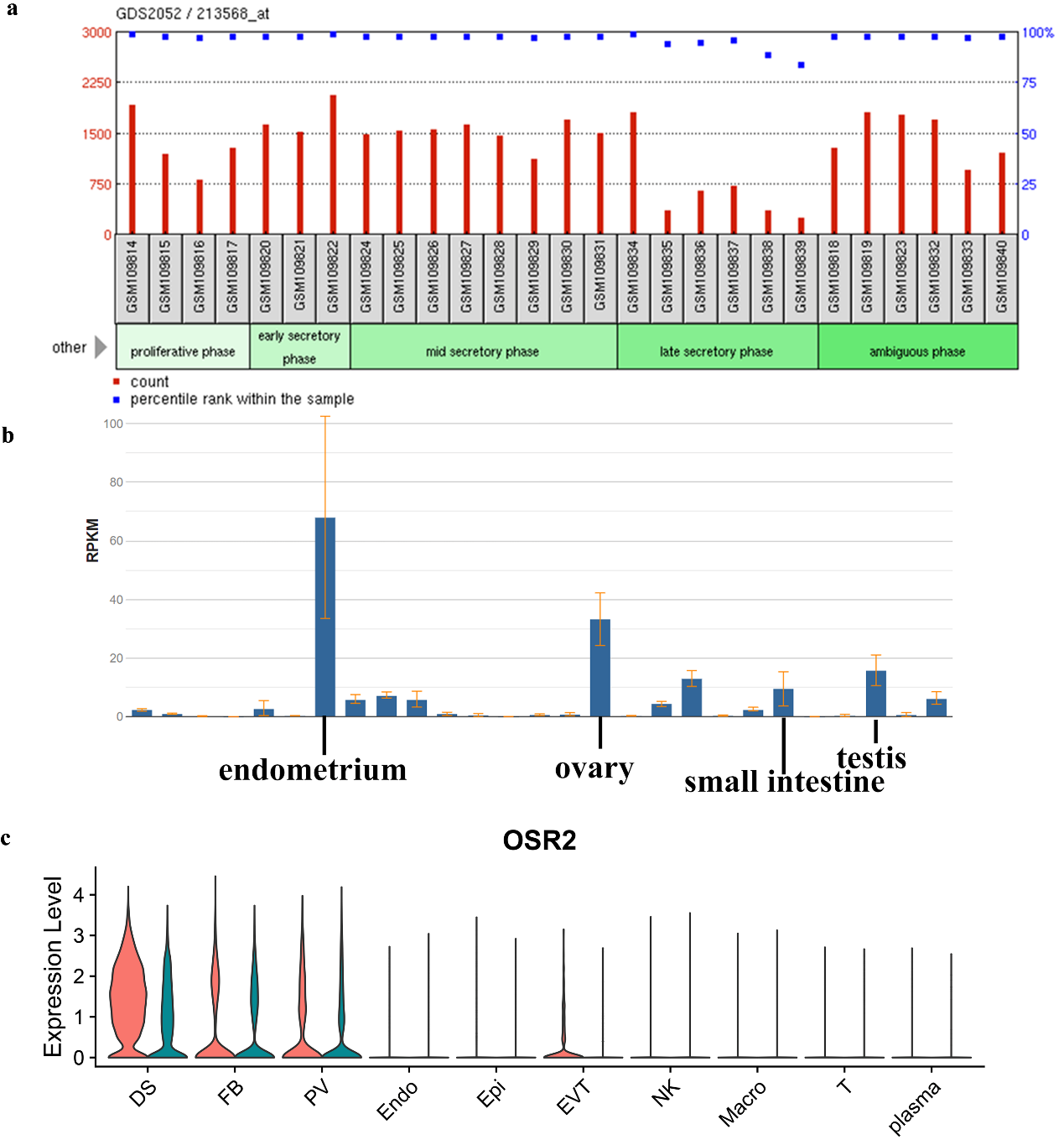


**Supplementary Fig S3.** Specifically high OSR2 expression in endometrium and decidual stromal cells of endometrium. **(a)** Gene Expression Omnibus (GEO) database indicates that odd-skipped related transcription factor 2 (OSR2) is abundantly expressed in different phases of menstrual cycles, especially in the mid-secretory phase (expression level higher than that of 90% of the genes) (Talbi, et al., 2006). The figure is used with permission from the publisher. **(b)** National Center for Biotechnology Information (NCBI) database suggests that OSR2 is most specifically and abundantly expressed in endometrium (Fagerberg, et al., 2014). The figure is used with permission from the publisher. **(c)** scRNA-seq analysis of OSR2 expression in different cell types of deciduas of women with RSA(n=6) and those with normal early pregnancies (n=5) by using VlnPlot functions in single cell R tool kit Seurat. DS: decidual stromal cells; FB: fibroblasts; PV: peripheral vascular cells; Endo: endothelial cells; Epi: epithelial cells; EVT: extravillous trophoblast cells; NK: natural killer cells; Macro: macrophages; T: T cells; plasma: plasma cells.


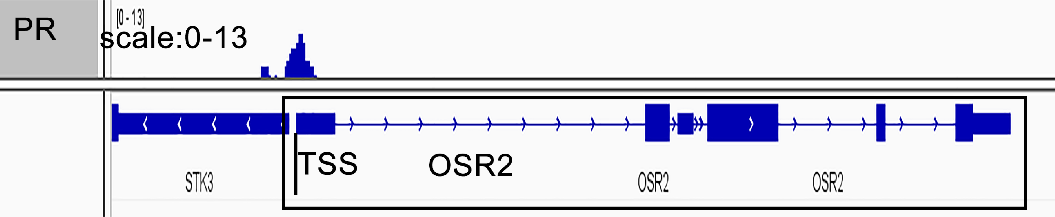


**Supplementary Fig S4.** The representative peaks for binding of PR (progesterone receptor) at the OSR2 promoter compared with input assayed by Chromatin immunoprecipitation sequencing (ChIP-seq) during HESCs decidulization (Kaya, et al., 2015). The figure is used with permission from the publisher. TSS: transcription start site.

**References**

Fagerberg L, Hallström BM, Oksvold P, Kampf C, Djureinovic D, Odeberg J, Habuka M, Tahmasebpoor S, Danielsson A, Edlund K et al. Analysis of the human tissue-specific expression by genome-wide integration of transcriptomics and antibody-based proteomics. Mol Cell Proteomics 2014; **13:** 397-406.

Kaya HS, Hantak AM, Stubbs LJ, Taylor RN, Bagchi IC, Bagchi MK. Roles of progesterone receptor A and B isoforms during human endometrial decidualization. Mol Endocrinol 2015; **29**:882-95

Talbi S, Hamilton AE, Vo KC, Tulac S, Overgaard MT, Dosiou C, Le Shay N, Nezhat CN, Kempson R, Lessey BA et al. Molecular phenotyping of human endometrium distinguishes menstrual cycle phases and underlying biological processes in normo-ovulatory women. Endocrinology 2006; **147:** 1097-1121.
